# Systematics and biology of *Cotesia typhae* sp. n. (Hymenoptera, Braconidae, Microgastrinae), a potential biological control agent against the noctuid Mediterranean corn borer, *Sesamia nonagrioides*

**DOI:** 10.3897/zookeys.682.13016

**Published:** 2017-07-05

**Authors:** Laure Kaiser, Jose Fernandez-Triana, Claire Capdevielle-Dulac, Célina Chantre, Matthieu Bodet, Ferial Kaoula, Romain Benoist, Paul-André Calatayud, Stéphane Dupas, Elisabeth A. Herniou, Rémi Jeannette, Julius Obonyo, Jean-François Silvain, Bruno Le Ru

**Affiliations:** 1 Laboratoire Evolution, Génomes, Comportement et Ecologie, UMR CNRS 9191, IRD 247, Université. Paris-Sud, Université Paris-Saclay, 1 Avenue de la Terrasse, 91198 Gif sur Yvette, France; 2 Canadian National Collection of Insects, 960 Carling Ave, K1A 0C6, Ottawa, Canada; 3 icipe: African Insect Science for Food and Health, Duduville Campus, Kasarani, P.O. Box 30772-00100, Nairobi, Kenya; 4 Institut de Recherche sur la Biologie de l’Insecte, UMR CNRS 7261, Université François-Rabelais de Tours, Faculté des Sciences, Parc Grandmont, 37200 Tours, France

**Keywords:** *Cotesia*, *Sesamia*, biological control, species complex, Africa, Mediterranean

## Abstract

Many parasitoid species are subjected to strong selective pressures from their host, and their adaptive response may result in the formation of genetically differentiated populations, called host races. When environmental factors and reproduction traits prevent gene flow, host races become distinct species. Such a process has recently been documented within the *Cotesia flavipes* species complex, all of which are larval parasitoids of moth species whose larvae are stem borers of Poales. A previous study on the African species *C. sesamiae*, incorporating molecular, ecological and biological data on various samples, showed that a particular population could be considered as a distinct species, because it was specialized at both host (*Sesamia nonagrioides*) and plant (*Typha domingensis*) levels, and reproductively isolated from other *C. sesamiae*. Due to its potential for the biological control of *S. nonagrioides*, a serious corn pest in Mediterranean countries and even in Iran, we describe here *Cotesia typhae* Fernandez-Triana **sp. n.** The new species is characterized on the basis of morphological, molecular, ecological and geographical data, which proved to be useful for future collection and rapid identification of the species within the species complex. Fecundity traits and parasitism success on African and European *S. nonagrioides* populations, estimated by laboratory studies, are also included.

## Introduction

Although the concept of species is questioned in situations characterized by a continuum of genetic differentiation and reproductive isolation between populations (The Marie Curie Speciation Network 2012), well described and identified species are still useful tools in many situations. For instance, in biological control the use of such species, with a defined host range and showing no gene flow with closely related species, limits the risk of confusion and guarantees the stability of its host range. The purpose of this paper is to describe a new species of parasitoid wasp, first considered as a host race of *Cotesia sesamiae* Cameron (Hymenoptera, Braconidae) (Branca et al. 2011; Kaiser et al. 2015). It is a potential candidate for the biological control of the Mediterranean maize stem borer, *Sesamia nonagrioides* (Lefèbvre, 1827) (Lepidoptera, Noctuidae).

*Cotesia* is one of the most diverse genera of the subfamily Microgastrinae (Hymenoptera, Braconidae), with almost 300 species already described (Yu et al. 2016), and probably over 1,000 species worldwide (e.g., Mason 1981). *Cotesia* was originally considered as a genus by Cameron in the 19^th^ century, and definitively split from the genus *Apanteles* by Mason (1981) in his generic reclassification of the Microgastrinae, wherein many *Apanteles* species were transferred to *Cotesia*. Microgastrine wasps are koinobiont endoparasitoids of lepidopteran larvae, and species attacking large larval hosts are often gregarious (Whitfield 1997, Quicke 2015). Females emit sex-pheromones that attract males and can mate upon emergence (Xu 2014). They locate their host at a distance and initiate oviposition upon recognition of chemical cues (Turlings and Fritzsche 1999, Jembere et al. 2003, Obonyo et al. 2010). The microgastrine wasps use a domesticated virus (called a bracovirus, Polydnaviridae) to inhibit the immune response of host larva. Bracoviruses are produced in the wasps’ ovaries by genes integrated in the wasp genome and injected in the host body together with the eggs (Asgari 2006, Gitau et al. 2007, Herniou et al. 2013). Within the host, the viral particles infect the host cells, which produce the viral proteins, which in turn inactivate the host immune cellular response and regulate the host metabolism to the benefit of wasp larvae (Herniou et al. 2013). Fully developed larvae egress from the host body and spin their cocoons to undergo metamorphosis. Host resistance processes can manifest at all these steps of the life cycle, among which encapsulation of the parasitoid eggs has been often reported and well described (Beckage 1998). Evolution of virulence mechanisms by the parasitoid may have driven the radiation of species within the genus *Cotesia* (Herniou et al. 2013).

The *Cotesia flavipes* species-group is a monophyletic complex made up of (until now) four allopatric sister species, all gregarious endoparasitoids of a few families of lepidopteran stem borers (Crambidae, Pyralidae, and Noctuidae) in monocot Poales (Poaceae, Typhaceae and Cyperaceae). The species-group comprises *Cotesia chilonis* (Munakata, 1912) from eastern Asia, including China, Japan and Indonesia; *Cotesia flavipes* (Cameron, 1891), from the Indian sub-continent, but also released and established in east Africa and the New World for the purpose of biological control; *Cotesia nonagriae* (Olliff, 1893), an Australian endemic recently removed from synonymy with *C. flavipes* (Muirhead et al. 2008, 2012), and *Cotesia sesamiae* (Cameron, 1906), from sub-Saharan and Southern Africa (Kimani-Njogu and Overholt 1997). Members of this species complex are economically important worldwide as biocontrol agents of cereals and sugarcane stem borer pests (Kfir et al. 2002, Lou et al. 2014, Mindigoyi et al. 2016, Polaszek and Walker 1991, Simões et al. 2012), and their presence in their native settings help regulate populations of important pests (Kfir et al. 2002, Liu et al. 2000).

Additional, cryptic species have been suspected within this complex and several papers have explored this possibility, especially in regard to *C. flavipes* (e.g., Muirhead et al. 2012) and *C. sesamiae*. In the latter species, studies made from samples collected in maize fields on a few pest species showed that local adaptation to host resources and environmental factors were major drivers of intra-species genetic diversity (Dupas et al. 2008, Gitau et al. 2007, 2010). Subsequently, Branca et al. (2011) analysed a large sample of *C. sesamiae* covering most of the species’ distribution area and a large range of host and plant species. They provided evidence that variations of host range were associated with sequence variation of a virulence gene, CrV1, which could be used as a marker of host races. Analysis of microsatellite markers revealed gene flow between the host races, except for one population specialized on the noctuid *Sesamia nonagrioides* (Branca et al. in prep.). One method to get an insight into the evolutionary stability of host-parasitoid associations is to characterize phylogenetic relationships between so-called host races. Kaiser et al. (2015) performed a phylogenetic analyses of the *C. sesamiae* samples based on mitochondrial, viral and non-viral nuclear markers, and demonstrated that the samples from the *S. nonagrioides* host race formed a highly supported monophyletic lineage showing all the hallmarks of a cryptic species. The authors confirmed the species status of this lineage by showing that it was reproductively isolated from the other lineages of *C. sesamiae* and from *C. flavipes*. Furthermore they showed that it was the only lineage being virulent against *S. nonagrioides*, and specifically so. Combined evidence for ecological specialization, selection for divergent host adaptation and for reproductive isolation, allowed them to conclude that this lineage was formed by ecological (adaptive) speciation. In addition, some morphological differences were readily identifiable.

Based on a wealth of information – morphological, molecular, biological, and ecological – we describe this new species of *Cotesia* from Africa, the fifth member of the *flavipes* complex, and present the first data showing that it is a successful parasitoid of European populations of *S. nonagrioides*, a major maize pest in West Africa and in Mediterranean countries.

## Materials and methods

### Morphological description

We studied 175 specimens from six different countries, representing ten populations from four out of the five known species within the *flavipes* complex (Table 1). We could not examine specimens of *Cotesia nonagriae*, but this Australian species has recently been redescribed and illustrated (Muirhead et al. 2008, 2012).

**Table 1.** Specimens studied for this paper. F- female specimen, M- male specimen.

Species	Country of origin	Collecting year	# of Specimens	Host caterpillar/host plant
*C. flavipes*	Trinidad	1972 & 1980	4 F, 3 M	*Diatraea lineolata* /unknown
*C. flavipes*	Colombia	1978	2 F, 7 M	Unknown/unknown
*C. flavipes*	Barbados	1977	2 F	Unknown/sugar cane
*C. flavipes*	India	1954	3 F	Unknown/unknown
*C. flavipes*	Kenya	2010	25 F, 5 M	*Chilo partellus*/maize
*C. sesamiae*	Kenya (Mombasa)	2010	25 F, 5 M	*Sesamia calamistis* /maize
*C. sesamiae*	Kenya (Kitale)	2012	25 F, 5 M	*Busseola fusca*/maize
*C. chilo*	Japan	2008	2 F, 2 M	Unknown/rice
*C. typhae* sp. n.	Kenya (Makindu)	2013	25 F, 5 M	*Sesamia nonagrioides*/ *Typha domingensis*
*C. typhae* sp. n.	Kenya (Kobodo)	2013	25 F, 5 M	*Sesamia nonagrioides*/ *Cyperus dives*

**Figure 1.**
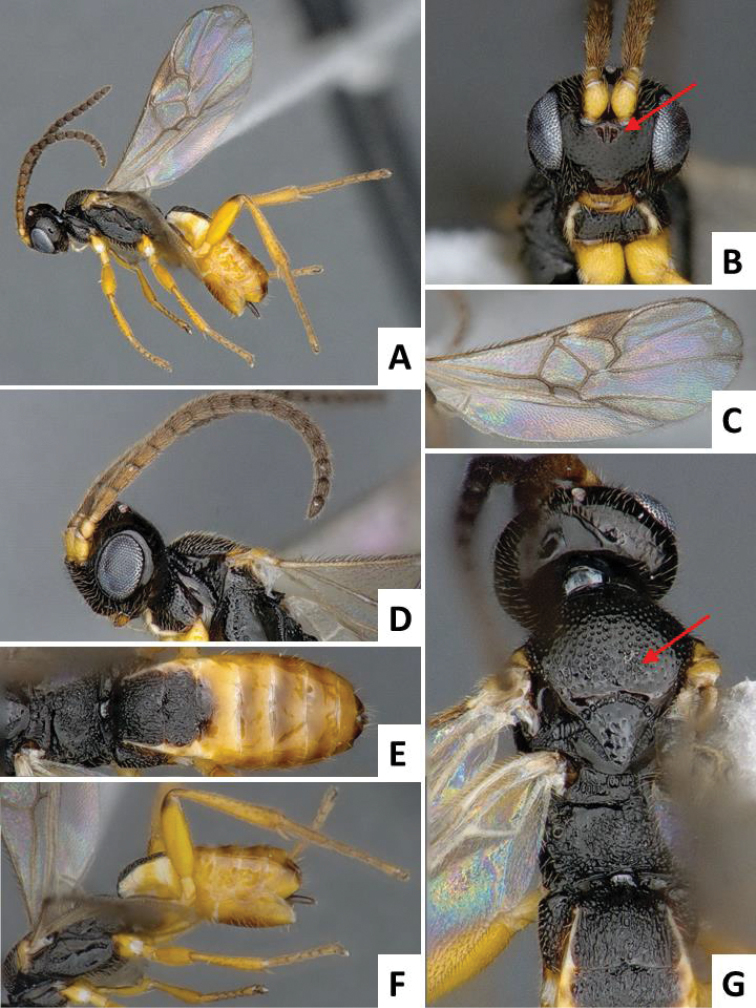
*Cotesia typhae*, holotype, female specimen from Makindu, Kenya. **A** Habitus, lateral view **B** Head, frontal view (arrow shows face projection between antennal base) **C** Wings **D** Head and mesosoma (partially), lateral view **E** Propodeum and metasoma, dorsal view **F** Mesosoma and metasoma, lateral view **G** Head, mesosoma and tergites 1-2, dorsal view (arrow shows anteromesoscutum punctures).

**Figure 2.**
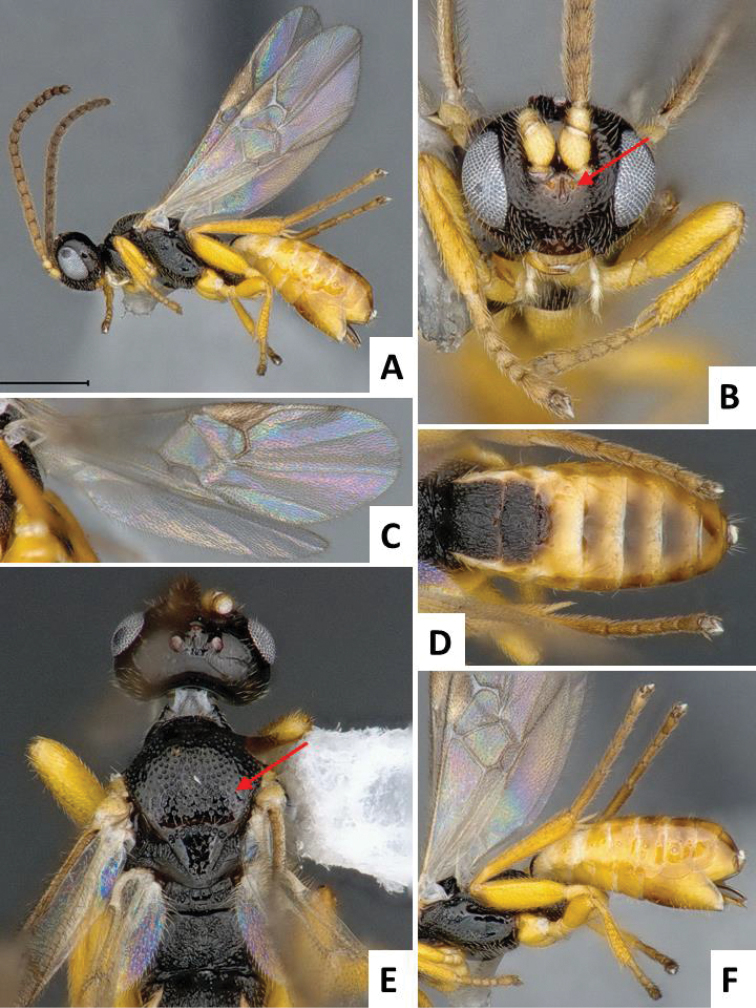
*Cotesia typhae*, paratype, female specimen from Kobodo, Kenya. **A** Habitus, lateral view **B** Head, frontal view (arrow shows face projection between antennal base) **C** Wings **D** Metasoma, dorsal view **E** Head and mesosoma, dorsal view (arrow shows anteromesoscutum punctures) **F** Metasoma, lateral view.

**Figure 3.**
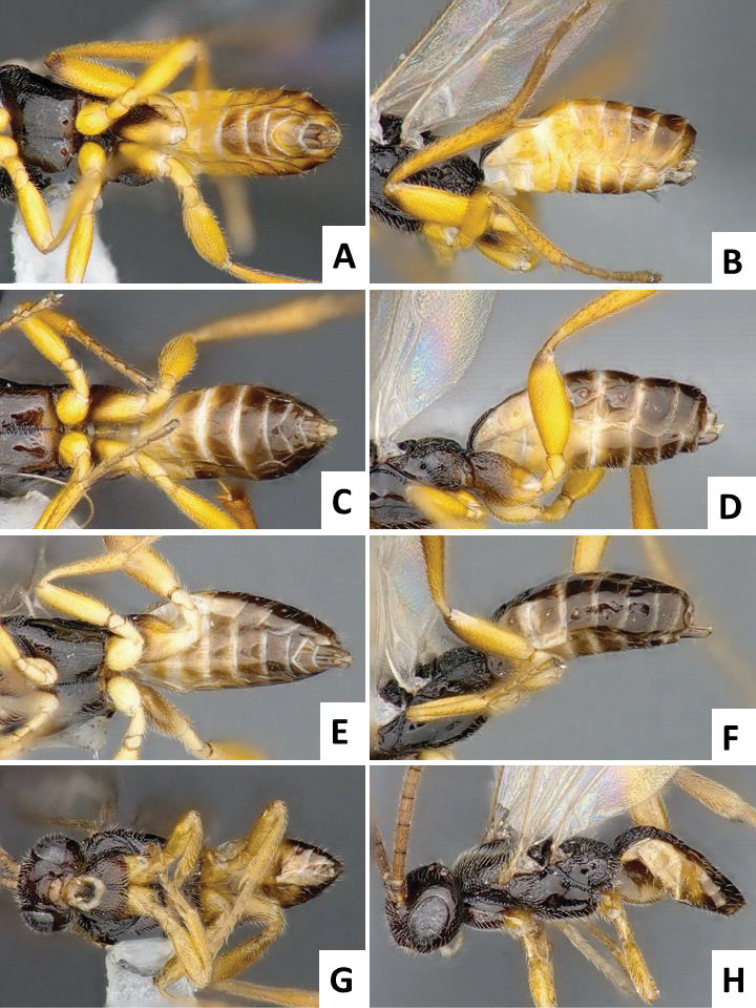
Male metasoma in ventral and lateral view. **A, B** *Cotesia typhae*, paratype specimen from Kenya **C, D** *Cotesia sesamiae*, specimen from Kenya **E, F** *Cotesia flavipes*, specimen from Kenya **G, H** *Cotesia chilonis*, specimen from Japan.

**Figure 4.**
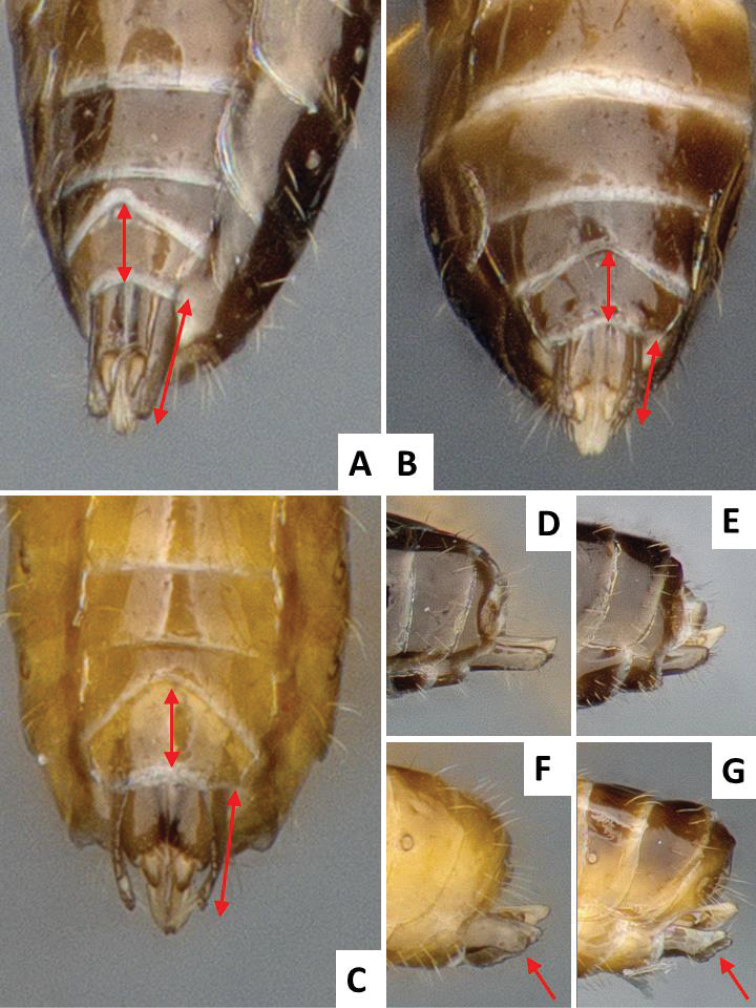
External male genitalia in ventral and lateral view; arrows show length of paramere and sternite 8. **A, D** *Cotesia flavipes* specimen from Kenya **B, E** *Cotesia sesamiae*, specimen from Kenya **C, F, G** *Cotesia typhae*, paratype specimen from Kenya.

**Figure 5.**
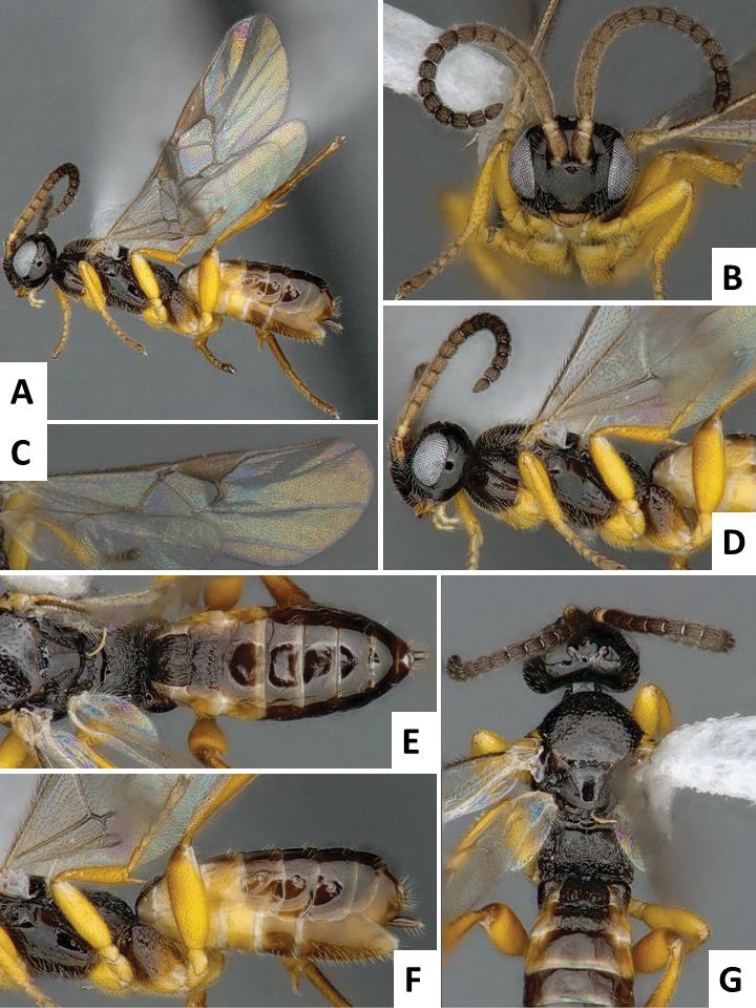
*Cotesia sesamiae*, female specimen from Kitale, Kenya. **A** Habitus, lateral view **B** Head, frontal view **C** Wings **D** Head and mesosoma, lateral view **E** Scutellar disc, propodeum and metasoma, dorsal view **F** Mesosoma and metasoma, lateral view **G** Head, mesosoma and tergites 1-4, dorsal view.

**Figure 6.**
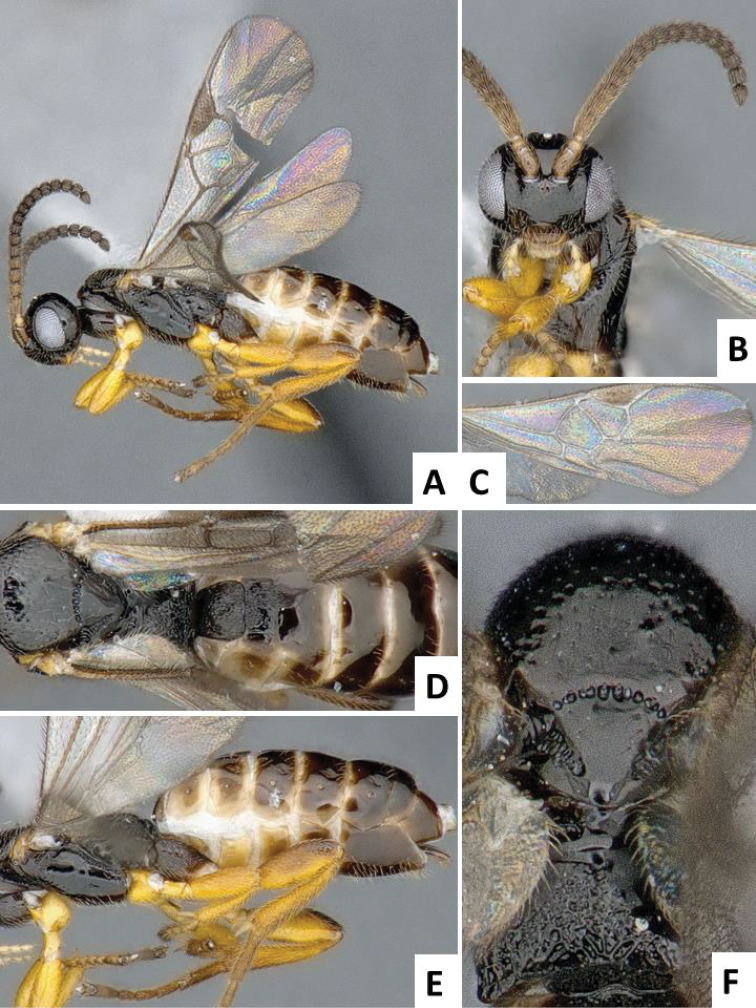
*Cotesia sesamiae*, female specimen from Mombasa, Kenya. **A** Habitus, lateral view **B** Head, frontal view **C** Wings **D** Mesosoma and metasoma (partially), dorsal view **E** Mesosoma and metasoma, lateral view **F** Anteromesoscutum, scutellar disc and propodeum, dorsal view.

**Figure 7.**
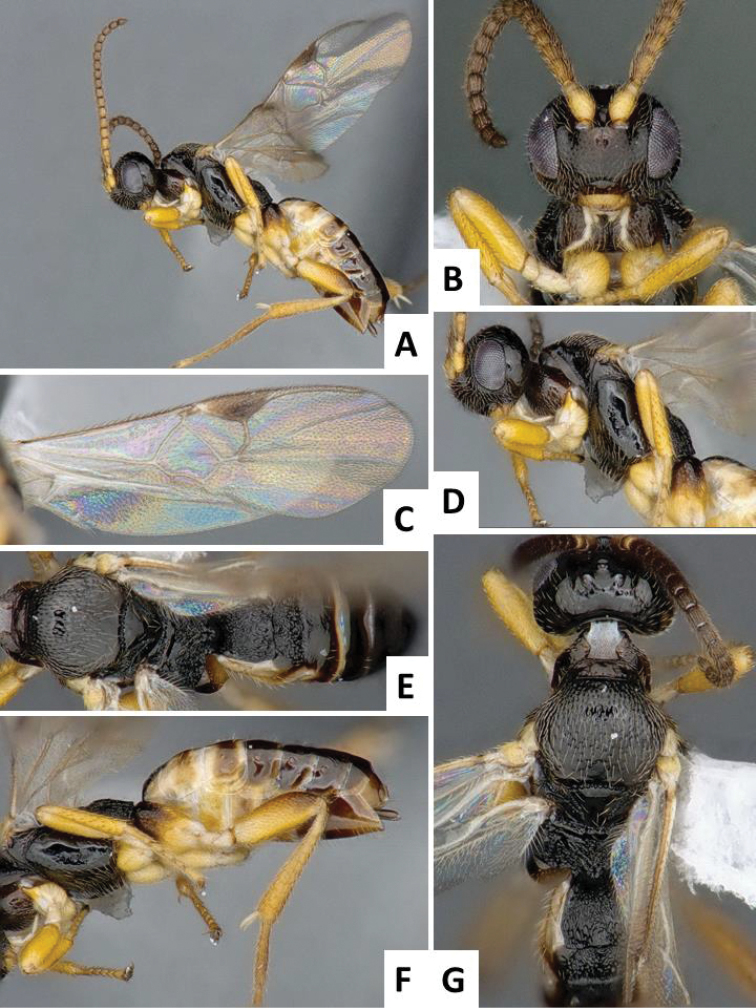
*Cotesia flavipes*, female specimen from Mombasa, Kenya. **A** Habitus, lateral view **B** Head, frontal view **C** Wings **D** Head and mesosoma, lateral view **E** Mesosoma and metasoma, dorsal view **F** Mesosoma and metasoma, lateral view **G** Head, mesosoma and tergites 1-2, dorsal view.

**Figure 8.**
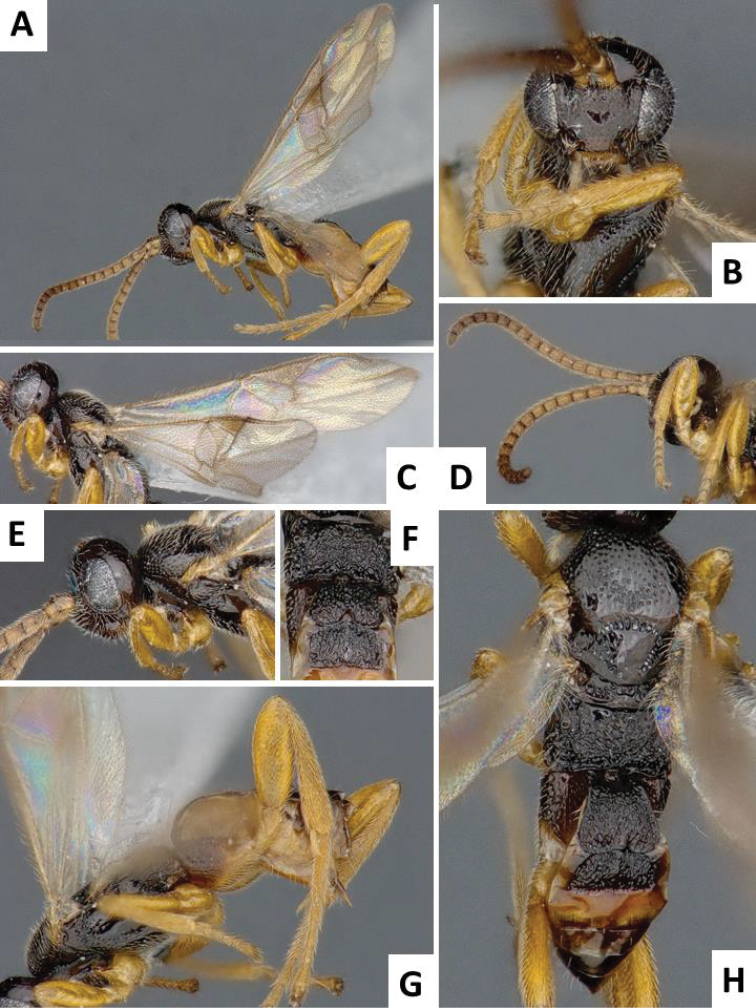
*Cotesia chilonis*, female specimen from Takatsuki, Japan. **A** Habitus, lateral view **B** Head, frontal view **C** Wings **D** Antennae, front and middle legs, lateral view **E** Head, lateral view **F** Propodeum, tergites 1-2, dorsal view **G** Mesosoma and metasoma, lateral view **H** Mesosoma and metasoma, dorsal view.

We evaluated a number of morphological characters proposed in previous studies (Kimani-Njogu and Overholt 1997, Muirhead et al. 2008), and others characters are explored for the first time in this species complex. Morphological terms and measurements of structures are mostly those used by Mason (1981), Huber and Sharkey (1993), Whitfield (1997), Karlsson and Ronquist (2012), and Fernandez-Triana et al. (2014). All characters used in this paper are illustrated in Figs 1–8.

In the species description, body ratios and measurement values are presented for the holotype first, followed by the range within the species in parentheses.

Photos were taken with a Keyence VHX-1000 Digital Microscope, using a lens with a range of 10–130 ×. Multiple images were taken of a structure through the focal plane and then combined to produce a single in-focus image using the software associated with the Keyence System. Plates were prepared using Microsoft PowerPoint 2010.

Institution acronyms used:

**CBGP** Centre de Biologie pour la Gestion des Populations, Montpellier, France.

**CNC** Canadian National Collection of Insects, Ottawa, Canada.

### Molecular characterization

In order to check the molecular-specific characterization of *Cotesia typhae*, we used the COI (*cytochrome oxydase I*) sequences from Kaiser et al. (2015) (listed in Appendix 1) to calculate the divergence between pairs of *Cotesia* species and populations. The divergence corresponds to the number of nucleotide differences divided by the total number of nucleotides. Since there are several samples for each species and population, the minimum and maximum divergence is given for all pairs.

### Distribution, ecology and abundance

Knowing that the new *Cotesia* species was found exclusively on *S. nonagrioides* on two plant families, Typhaceae and Cyperaceae (Kaiser et al. 2015), its distribution, ecology and abundance are characterized here from a collection of *S. nonagrioides* larvae on these two plant families, collected in 13 countries in sub-Saharan Africa between 2004 and 2013. *Sesamia nonagrioides* larvae were sampled from wild plants on banks of streams or rivers and in swamps, the favorite habitat of this species, which is rarely recorded from maize in East Africa. Plants were carefully inspected for stem borer infestations. Symptoms of infestation included scarified leaves, dry leaves and shoots (dead hearts), frass or holes bored. Infested plants were cut and dissected in the field; larvae collected were reared on an artificial diet (Onyango and Ochieng-Odero 1994) until pupation or emergence of parasitoid larvae. *Sesamia nonagrioides* were identified at the adult stage by dissection of the genitalia. After emergence, adult *Cotesia* were stored in absolute ethanol and identified by genotyping CrV1 sequence.

### Life history traits and parasitoid success in European host populations

**Insect material**

The *C. typhae* laboratory-reared strains were collected initially from Kenya localities (Kobodo: 0.68°S; 34.41°E or Luanda: 0.48°S; 34.30°E, depending on the availability of the strains). They were reared on a Kenyan *S. nonagrioides* strain (collected initially from Makindu: 2.28°S; 37.82°E), according to the method described by Overholt et al. (1994). Parasitoid success was tested on this Kenyan strain and on two *S. nonagrioides* European strains collected respectively in France (Longage, 43.37°N; 1.19°E) and Italy (Monterotondo scalo, 42.06°N; 12.60°E). The Kenyan and French strains were reared as described above. The Italian larvae were sent from the University of Perugia.

**Longevity experiments**

Clusters of cocoons were each placed in a 0.5L disposable plastic box with a 1.5 cm diameter opening clogged with a foam cork. One of the three following food sources was placed in the box to test their effects on longevity: honey droplets and a tap water-imbibed cotton ball; a cotton ball imbibed with a 2% saccharose solution or a 20% solution. These small cages were placed at 21°C, with internal relative humidity around 75%. Dead insects were counted every day for the 2% sugar solution and at least every two days for the two other food sources, from 24h following emergence.

**Realized fecundity**

One-day-old wasps were taken from the cages as above and allowed to oviposit in one host larva per day, for four days. Parasitized larvae were kept individually in Petri dishes (2 cm high) with approximately 10cm^3^ piece of diet, until emergence of the parasitoid larvae or pupation. The diet was replaced by a piece of toilet paper 12 days after parasitism to facilitate cocoon formation.

**Parasitoid success**

Four weeks after hatching, i.e. when reaching the 5^th^- 6^th^ stadium, larvae were exposed each to one wasp, then kept fed with the diet, in the conditions described above, until emergence of the parasitoid larvae, or pupation. Recorded traits are specified in Table 8. Individual cocoon weight was calculated by dividing the weight of the cocoon cluster by the number of emerged adults and dead nymphs.

**Data analyses**

Kaplan Meyer tables from XLSTAT were used to estimate daily mortality and median longevity. The procedure included three tests of equality of the survival curves (Wilcoxon, Log-rank and Tarone-War) that gave identical P-values, so only Wilcoxon’s result is given in this study. Comparisons of traits of parasitoid success on the three host strains were performed with the R package. As some of the traits did not follow a normal distribution (Shapiro statistic) or did not fulfill homoscedasticity (Bartlett statistic), the Kruskal-Wallis statistic was used to compare the quantitative traits recorded for the three host strains, followed by the Dunn post-hoc multiple comparison test. Chi-square was used to compare the issue of parasitism. Sample sizes are given in Table 8. The percentage of females in the cluster was not included in the analyses when pupal mortality was equal to or exceeded 30%. This occurred for 11 clusters obtained from the French host strain and two clusters obtained from the Kenyan strain.

## Results

### Morphological study

#### Cotesia typhae

Taxon classificationAnimaliaHymenopteraBraconidae

Fernandez-Triana sp. n.

http://zoobank.org/EC4B19D5-9087-4698-A67D-E53EAE5E532E

Figs 1 , 2

##### Holotype.

Female (CBGP).

##### Type locality.

Kenya, Makindu, 2.28°S, 37.82°E.

##### Holotype label details.

Kenya, Makindu, xi.2010, ex *Sesamia nonagrioides* on *Typha domingensis* Pers. Voucher code: CNC634434. Other code on label: F78.

##### Paratypes.

CBGP, Montferrier s/Lez, France; CNC, Canada; International Centre of Insect Physiology and Ecology, Nairobi, Kenya; Natural History Museum London, UK; Smithsonian National Museum of Natural History, Washington DC, USA. 24 female, 5 male specimens, same locality as holotype; 25 female, 5 male specimens from Kenya, Kobodo, 0.41°S, 34.25°E. iii.2013, ex *Sesamia nonagrioides* on *Cyperus dives* Delile.

##### Previous records.

This species has been referred to as the *C. sesamiae* population, harbouring Cs Snona haplotype on CrV1 locus (Branca et al. 2011), as the *C. sesamiae* lineage 2 analysed by Kaiser et al. (2015) and as the sample CsBV G4675 sequenced for 3 viral genes in Jancek et al. (2013).

##### Diagnosis.

The new *Cotesia* is relatively distinct from other members of the *flavipes* complex (Table 2). The most distinctive diagnostic characters are the median projection present between the base of the antennae, the punctures on the anteromesoscutum, the length and shape of the paramere, and the relative length of the antennal flagellomere. The median projection between the base of the antennae is depressed (compared to the rest of the face), usually paler than the rest of the face, and has a strongly excavated median longitudinal sulcus (Figs 1B, 2B); all other species within the *flavipes* complex have a less depressed median projection on the face, usually the same color (or at most slightly lighter) as the rest of the face, and the median sulcus is not defined (*nonagriae*) or is less strongly excavated (Figs 5B, 6B, 7B, 8B). The anteromesoscutum punctures (Figs 1G, 2E) are the largest, densest, and most widely distributed (present near the posterior margin of the anteromesoscutum) among all species within the *flavipes* complex (compare against Figs 5G, 6F, 7G, 8H). The paramere length (Figs 3A, B, 4C, F, G) is intermediate compared to the other species (longer than in *chilonis/sesamiae* and shorter than in *flavipes/nonagriae*; compare Figs 3D, F, H, 4D, E), and its shape seems to be distinctive, with a somewhat widened part near the apex (Fig. 4F, G). The antennal flagellomeres (Figs 1A, D, 2A, B) are the longest among the entire *flavipes* complex (compare versus Figs 5A, B, D, 6A, B, 7A, B, 8A, D). The color of metasoma laterally and ventrally (laterotergites, sternites and hypopygium) is light yellow-orange (Figs 1A, F, 2A, F). This character is useful in recognizing *typhae*, at least in Africa, as all other *Cotesia* species within this complex generally have a much darker metasoma latero-ventrally (e.g. Figs 5A, F, 6A, E, 7A, F, 8G); however, some populations of *C. flavipes* we have examined have a light-colored metasoma, so this character is not absolutely diagnostic.

**Table 2.** Diagnostic characters within the *Cotesia flavipes* complex. Data on host caterpillar species from Branca et al. (2011), Muirhead et al. (2012), Sallam (2006), and Kaiser et al. (2015).

	*Cotesia chilonis*	*Cotesia flavipes*	*Cotesia nonagriae*	*Cotesia sesamiae*	*Cotesia typhae*
**Scutoscutellar sulcus**	Straight (Fig. 8H)	Curved (Fig. 7E)	Curved	Curved (Fig. 5F)	Curved (Figs 1G, 2E)
**Antero-mesoscutum (AMS) punctures**	Large punctures (diameter larger than distance between punctures) in most of AMS, including most of the posterior half (Fig. 8H)	Relatively small punctures on anterior half of AMS, posterior half almost entirely smooth (Fig. 7E, G)	Relatively small punctures on anterior half of AMS, posterior half almost entirely smooth	Relatively small punctures on anterior half of AMS, posterior half almost entirely smooth (Figs 5G, 6F)	Large punctures (diameter larger than distance between punctures) in most of AMS, including most of the posterior half (Fig. 2G, E)
**Face projection between antennal base**	Acute, triangular projection with clearly impressed median longitudinal sulcus (Fig. 8B)	Acute projection (sometimes projection less acute, margin almost straight) with clearly impressed median longitudinal sulcus (Fig. 7B)	More or less straight margin, with no clearly impressed, median longitudinal sulcus	Acute projection (sometimes projection less acute, margin almost straight) with clearly impressed median longitudinal sulcus (Figs 5B, 6B)	Acute, triangular projection with clearly impressed median longitudinal sulcus (Fig. 1B, B)
**Paramere length (observed externally, without removing genitalia from specimen)**	Short, around 1.0 × as long as median length of sternite 8 (partially visible in Fig. 3G, H)	Large, clearly more than 1.5 × (usually up to 2.0x) as long as median length of sternite 8 (Fig. 4A, D)	Large, clearly more than 1.5 × (usually up to 2.0x) as long as median length of sternite 8	Short, around 1.0 × as long as median length of sternite 8 (Fig. 4b, E)	Relatively large, around 1.5 × as long as median length of sternite 8 (Fig. 4C)
**Paramere shape**	Rather uniformly narrowing from base to rounded apex	Rather uniformly narrowing from base to rounded apex (Fig. 4D)	Rather uniformly narrowing from base to rounded apex	Rather uniformly narrowing from base to rounded apex (Fig. 4E)	With a broad, widened area near apex (Fig. 4F, G)
**Antennal flagellomeres**	Relatively short (3+ about as long as wide)	Relatively short (2+ about as long as wide)	Relatively short (2+ about as long as wide)	Relatively short (3+ about as long as wide)	Relatively long (1–4 much longer than wide)
**Natural known hosts**	*Chilo supressalis, C. partellus* (Crambidae)	More than 7 species (Crambidae & Noctuidae)	*Bathytricha truncata* (Noctuidae)	More than 34 species (mostly Noctuidae & Crambidae)	*Sesamia nonagrioides* (Noctuidae)

##### Description.

Head and mesosoma mostly dark brown to black (except for scape, pedicel, wing base and tegula yellow; antennal flagellomeres brown; mandibles and labrums orange-yellow, and face projection between antennal base usually light brown); legs mostly yellow (except for metafemur with brown dorsal tip on posterior 0.1, and metatarsus light brown to brown); metasoma mostly yellow-brown to yellow-orange (except for mediotergites 1 and 2 dark brown to black, and mediotergites 3+ usually with brown spot centrally, near anterior margin). Wings with veins mostly brown, pterostigma brown with pale spot on anterior 0.3.

Head wider than high; face with acute, triangular projection between antennal base, the projection with clearly impressed median longitudinal sulcus; head dorsally smooth; gena laterally and dorsally as wide or wider than eye width; anteromesoscutum with relatively deep, coarse and large punctures (puncture diameter larger than distance between punctures), puncture density similar on most of the anteromesoscutum, including posterior half; scutoscutellar sulcus strongly curved, with 10-12 impressions; scutellar disc mostly smooth, with shallow and sparse punctures; propodeum mostly sculptured with an irregular pattern of strong carinae; mediotergites 1-2 mostly covered by strong longitudinal striae, mediotergites 3+ mostly smooth; hypopygium relatively small, apical tip in lateral view shorter than apical tip of tergites; paramere with broad, widened area near apex; paramere relatively large, around 1.50 × as long as median length of sternite 8.

*Body ratios.* Length of flagellomere 2/length of flagellomere 14: 1.71 × (1.50–1.86). Metafemur length/width: 3.06 × (2.92–3.25). Length of inner spur of metatibia/length of first segment of metatarsus: 0.48 × (0.46–0.52). Length of inner spur of metatibia/length of outer spur of metatibia: 1.07 × (1.07–1.18). Pterostigma length/width: 2.81 × (2.61–2.88). Length of fore wing vein r/length of fore wing vein 2RS: 0.82 × (0.82–1.00). Mediotergite 1 length/mediotergite width at posterior margin: 1.07 × (0.93–1.20). Length of mediotergite 2/length of mediotergite 3: 0.89 × (0.83–1.00).

*Body measurements* (all in mm). Body length: 2.40 (2.20–2.50). Fore wing length: 2.10 (2.10–2.20). Length of antennal flagellomere (F), F1: 0.15 (0.14–0.17), F2: 0.12 (0.12–0.13), F3: 0.11 (0.10–0.11), F14: 0.07 (0.06–0.08), F15: 0.07 (0.06–0.08), F16: 0.10 (0.09–0.11). Metafemur length: 0.55 (0.51–0.56). Metafemur width: 0.18 (0.16–0.19). Metatibia length: 0.71 (0.66–0.74). First segment of metatarsus length: 0.31 (0.28–0.31). Length of inner spur of metatibia: 0.15 (0.13–0.16). Length of outer spur of metatibia: 0.14 (0.11–0.14). Ovipositor sheaths length: 0.18 (0.15–0.18). Pterostigma length: 0.45 (0.145–0.49). Pterostigma width: 0.16 (0.16–0.18). Length of fore wing vein r: 0.09 (0.09–0.11). Length of fore wing 2RS: 0.11 (0.10–0.12). Length of mediotergite 1: 0.30 (0.27–0.31). Width at posterior margin of mediotergite 1: 0.28 (0.25–0.32). Length of mediotergite 2: 0.16 (0.14–0.20). Length of mediotergite 3: 0.18 (0.15–0.20).

##### Etymology.

Named after the main host plant on which the wasp parasitizes its host caterpillar, Kaiser et al. (2015).

##### Notes.

*Cotesia typhae* occurs sympatrically with *C. sesamiae* and *C. flavipes* (the latter introduced into Africa). Among these three species, *typhae* is the largest (body and fore wing lengths usually 0.2–0.3 mm longer than the two others), it also has a more sculptured anteromesoscutum and a longer antenna (especially flagellomeres 1–4 which are significantly longer).

### Molecular characterization

Between species, pairwise divergence of COI sequences ranged from 2.6% to 4.2%, and distances observed between *C. typhae* and the other *C. sesamiae* species fell in this range Table 3). Within species, divergence was close to zero for *C. typhae*, *C. chilonis* and *C. flavipes*, and ranged from zero to 2.8% in *C. sesamiae*. The higher within-species values in *C. sesamiae* are explained by the divergence between the Kitale and Mombassa populations, reflecting their affiliation with different lineages, as shown by Kaiser et al. (2015) (Table 4).

**Table 3.** Minimum and maximum divergence of COI sequences between all pairs of species.

	*C. typhae*	*C. sesamiae*	*C. flavipes*	*C. chilonis*
*C. typhae*	0–0.002			
*C. sesamiae*	0.026–0.035	0–0.028		
*C. flavipes*	0.033–0.035	0.031–0.042	0	
*C. chilonis*	0.035	0.030–0.037	0.037	0

**Table 4.** Minimum and maximum divergence of COI sequences between *C. typhae* and two populations of *C. sesamiae*.

	*C. typhae*	*C. sesamiae* Kitale	*C. sesamiae* Mombasa
*C. typhae*	0–0.002		
*C. sesamiae* Kitale	0.03–0.035	0–0.014	
*C. sesamiae* Mombasa	0.026–0.03	0.019–0.028	0–0.003

### Distribution, ecology and abundance

Among the ten sampled countries and 65 sampled localities hosting *S. nonagrioides* on Typhaceae and Cyperaceae, larvae parasitized by *C. typhae* were found in the three most sampled countries (highest numbers of localities and collected larvae), Ethiopia, Kenya and Tanzania (Table 5), in a total of 12 localities (Table 6). This showed that the probability of discovering *C. typhae* depended on the sampling effort, so this species may well be present in other sub-Saharan Africa areas inhabited by *S. nonagrioides* (Kergoat et al. 2015).

**Table 5.** Presence of *Cotesia typhae* in the sampled countries. Results of collections of *S. nonagrioides* in sub-Saharan Africa from 2004 to 2013. For each country the Table shows the number of localities containing Typhaceae and Cyperaceae plants, the total number of *S. nonagrioides* larvae collected there during the period, and whether some were parasitized by *C. typhae*.

Country	Number of sampled localities with Typhaceae & Cyperaceae	Number of *S. nonagrioides* larvae	presence of *Cotesia typhae*
Benin	1	26	no
Botswana	1	2	no
Cameroun	1	1	no
Ethiopia	5	167	YES
Kenya	26	1253	YES
R. Congo	2	38	no
R.D.C.	2	26	no
Rwanda	1	7	no
Tanzania	18	463	YES
Tanzania, Pemba	1	1	no
Tanzania, Zanzibar	3	25	no
Uganda	4	26	no

We then estimated the percentage of parasitized *S. nonagrioides* in the localities where the parasitoid was present. It varied from less than five to more than 70 % (Table 6), with a mean value of 20.3 % (standard error 4.0 %, n=18). All values, except the highest, ranged between 3.4 and 33.3% of parasitized larvae. Among the 660 parasitized larvae, 5 were parasitized by *Cotesia* other than *C. typhae* (4 *C. sesamiae* and 1 *C. flavipes*). Repeated findings of *C. typhae* in different years in the same locality, as seen in two Kenyan localities (Mbita Lwanda, 4 collections over 9 years; Makindu, 3 collections over 4 years), showed that locality and plant-host combination were good criteria for finding this new species.

**Table 6.** Percentage of parasitism of *S. nonagrioides* larvae in the localities where *C. typhae* was found.

Country	Locality	Latitude / Longitude	EDate	Plant species	Nbr *S. n.* larvae	% parasitism
ETHIOPIA	Awasa	7.05°N, 38.47°E	Nov.-04	*T. domingensis*	64	6.3%
ETHIOPIA	Chamoleto	5.93°N, 37.53°E	Nov.-04	*T. domingensis*	16	18.8%
ETHIOPIA	Omolante	6.16°N, 37.67°E	Nov.-04	*T. domingensis*	27	22.2%
KENYA	Kabuto	0.35°S, 34.96°E	May-12	*C. dives*	6	33.3%
KENYA	Kobodo	0.86°S, 34.57°E	March-13	*C. dives*	42	7.1%
KENYA	Makindu	2.28°S, 37.82°E	Nov.-10	*T. domingensis*	65	10.8%
KENYA	Makindu	2.28°S, 37.82°E	Feb.-11	*T. domingensis*	64	4.7%
KENYA	Masimba	2.15°S, 37.58°E	Dec.-06	*T. domingensis*	10	30.0%
KENYA	Masimba	2.15°S, 37.58°E	Apr.-08	*T. domingensis*	13	15.4%
KENYA	Mbita Lwanda	0.89°S, 34.67°E	Feb.-05	*T. domingensis*	68	27.9%
KENYA	Mbita Lwanda	0.89°S, 34.67°E	Oct.-08	*T. domingensis*	147	10.2%
KENYA	Mbita Lwanda	0.89°S, 34.67°E	June-07	*T. domingensis*	18	72.2%
KENYA	Mbita Lwanda	0.89°S, 34.67°E	March-13	*T. domingensis*	59	8.5%
KENYA	Rabuor	0.43°S, 34.91°E	March-13	*C. dives*	10	20.0%
KENYA	Rabuor	0.43°S, 34.91°E	March-13	*T. domingensis*	6	33.3%
KENYA	Sori	0.97°S, 34.28°E	March-13	*T. domingensis*	13	7.7%
TANZANIA	Arusha	3.37°S, 36.87°E	July-04	*T. domingensis*	29	3.4%
TANZANIA	Ruvu	6.70°S, 38.71°E	March-07	*C. exaltatus*	3	33.3%

### Life history traits and parasitoid success on different host strains

**Adult longevity**

**Figure 9.**
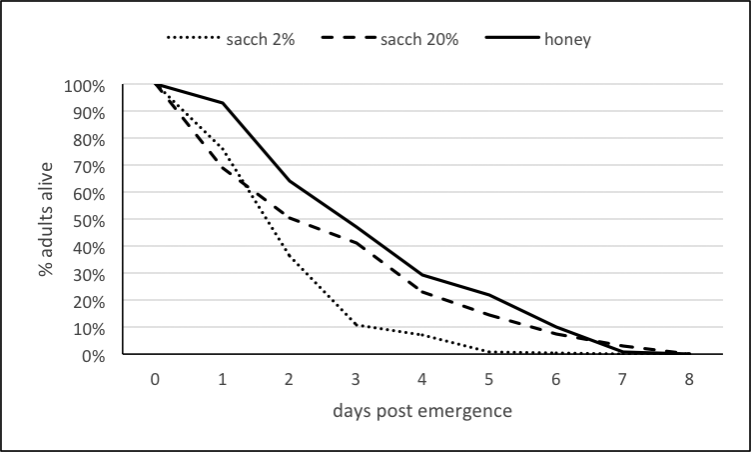
Survival curves of *C. typhae* adults fed honey (number of wasps: n=497), or 20% (n=742) or 2% (n=534) saccharose solutions in collective cages at 21°. Median lifespan is the time value observed at 50% survival.

The median longevity was close to three days when adults were fed honey, but equal to two days or less when they were fed with 20% or 2% saccharose solution respectively (Fig. 9). The survival curves were significantly different (W_2df_ = 129.78; *P* < 10^-4^). They showed that about 90% adults were dead six days after emergence when fed honey or 20% saccharose, and three days after emergence when fed 2% saccharose (Fig. 9).

**Realized fecundity**

**Figure 10.**
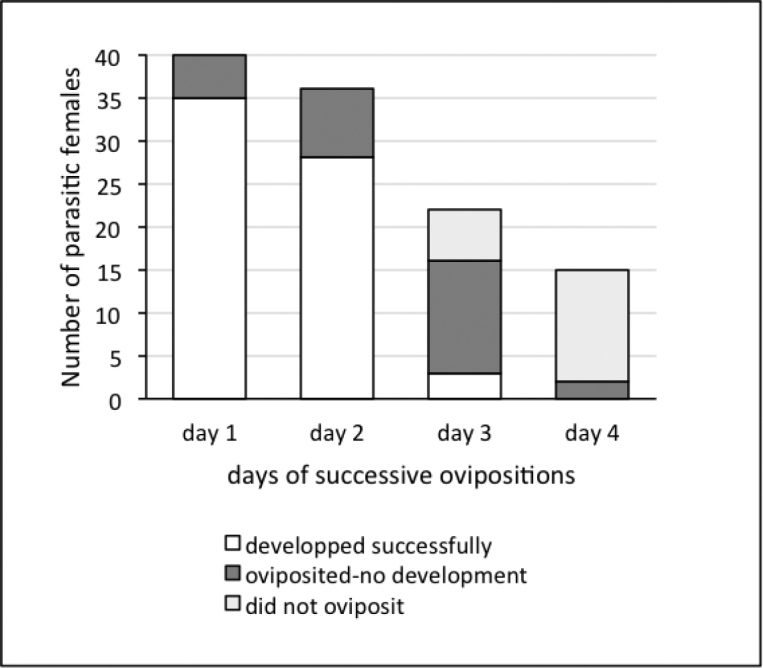
Issue of successive presentations of host larvae to *C. typhae* (one host per day for four days or less for wasps that died).

Females were given the opportunity to parasitize a maximum of four larvae, but they actually parasitized a mean number of only 2.3 larvae (Table 7), either because they died before the end of the experiment (almost half of them were dead on the third day, Fig. 10), or because they refused to oviposit, as observed for a few females on day 3, and for most of the surviving ones on day 4 (Fig. 10). About 2/3 of the stung larvae allowed successful parasitoid development (Table 7). Finally, females produced about 100 offspring during their lifetime, from two host larvae.

**Table 7.** Realized fecundity of *C. typhae* on Kenyan *S. nonagrioides*.

	adult lifetime (days)	stung larvae (nbr)	successfully parasitized larvae (nbr)	Offspring (total nbr)
Mean (N=40)	2.83	2.3	1.63	102.93
Standard error	1.17	0.11	0.11	6.2

**Table 8.** Development of *C. typhae* in Kenyan and European hosts. Bold characters indicate significant differences between host strains.

*S. nonagrioides* populations:	Kenya	France	Italy	Statistical analyses
*N: Nbr parasitized host larvae*	*58*	*58*	*47*	–
Host larval weight at time of parasitism (mg)	295 ± 11	272 ± 12	283 ± 12	KW_2df_ = 1.28 *P* = 0.331
% successful parasitism % host pupae % host larva mortality	69.0 (b) 12.1 19.0	67.2 (b) 13.8 19.0	89.4 (a) 2.1 8.5	**χ^2^_2df_ = 7.95 *P* = 0.019**
*N: Nbr of cocoon clusters analyzed below*	38	32	33	–
*Cotesia* larval development	14.2 ± 0.4 b	14.4 ± 0.2 b	12.9 ± 7.1 a	**KW_2df_ = 18.29 *P* = 10^-4^**
*Cotesia* pupal development (days)	8.2 ± 0.3 b	6.8 ± 0.2 a	7.1 ± 0.1 a	**KW_2df_ = 19.60 *P* < 10^-4^**
Cocoon number	60.3 ± 4.6 b	75.0 ± 5.5 a	64.6 ± 4.5 ab	**KW_2df_ = 7.67 *P* = 0.022**
Individual cocoon weight (mg)	1.3 ± 0.04	1,3 ± 0.05	1,2 ± 0.02	KW_2df_ = 4.20 *P* = 0.122
% *Cotesia* pupal mortality	10.4 ± 2.7 a	25.8 ± 4.6 b	3.0 ± 0.7 a	**KW_2df_ = 16.54 *P* < 10^-3^**
% females in the cluster	43.9 ± 5.4 c	35.0 ± 8.2 b	72 ± 3.8 a	**KW_2df_ = 17.98 *P* < 10^-3^**
Estimated Reproductive Rate (expected viable adults/mother)	37	37	56	–

In the next experiment, the possibility for *C. typhae* to develop in European populations was estimated by the incidence of the first oviposition, which ensured more than half of the wasp’s reproductive success.

**Parasitoid success in European host populations**

Susceptibility of European *S. nonagrioides* strains to the parasitoid was equal or even higher than that of the Kenyan strain, with for instance almost 90% of successfully parasitized Italian larvae. Several other traits differed between the host strains, with a trend for better performances in the Italian strain, which ranked “a” for the five progeny traits showing significant differences: faster larval and pupal development, resulting in a development time of 20 days; high offspring number per cluster, showing the lowest pupal mortality and highest ratio of females. Highest immature developmental time (22 days) was observed in the Kenyan host strain, and highest pupal mortality and lowest female ratio was observed in the French strain. From these traits, it is possible to estimate a reproductive rate, i.e. the expected number of viable adults per mother, by multiplying the proportion of successful parasitism (probability of host larvae successfully parasitized) by the mean number of produced cocoons and by the proportion of viable adults (1-proportion of pupal mortality). This approach indicated that a female *C. typhae* would produce 56 viable offspring from a host larva of the Italian population, and only 37 from the host larvae of the French or Kenyan populations. As discussed hereafter, most differences could be explained by the effect of rearing conditions on host larvae quality.

## Discussion

The morphological analysis conducted in this study, as well as the divergence of the CO1 sequences, confirmed the species status of the *C. sesamiae* lineage specialized on the noctuid *S. nonagrioides*. The CO1 divergence fell within the range of values observed between species of the *flavipes* complex. Morphological traits differentiated in this lineage included those used to distinguish species of the *flavipes* complex. This constitutes evidence for the existence of a fifth species in the *flavipes* complex. We named this new species *C. typhae*, based on the main host plant where it is found on its host. Whereas the first four species are allopatric in their endemic range, *C. typhae* is sympatric with *C. sesamiae* and may have differentiated from this species through divergent selection for adaptation on *S. nonagrioides* in Typhaceae and Cyperaceae, a permanent resource, and divergent selection for reproductive isolation (possibly facilitated by *Wolbachia*) (Kaiser et al. 2015).

It is likely that more species may be found in this complex. For instance, a relatively large CO1 divergence was also observed between *C. sesamiae* populations from Kitale (inland Kenya) and Mombassa (coastal Kenya), which are two host races with limited gene flow due to *Wolbachia* infection (Mochiah et al. 2002). Additional studies on the phylogenetic and biological relationships among those lineages, in particular the strength of bidirectional cytoplasmic incompatibilities related to *Wolbachia* strains, may reveal, in the future, the presence of an additional species.

Male genitalia were one of the differentiated morphological traits. This explains mating abnormalities observed by Kaiser et al. (2015) when crossing males of *C. typhae* with females of *C. sesamiae*, i.e. difficulties of males to disengage from females. It is one component of pre-zygotic barriers. In most animal species with internal fertilization, male external genitalia are the most rapidly evolving organs and are usually the first organs to diverge morphologically following speciation (Eberhard 2010; Yassin and Orgogozo 2013). Because of their rapid evolution and species-specificity, their illustration is a common feature in taxonomic literature to discriminate closely related species, particularly in insects (Yassin and Orgogozo 2013), including species of the *flavipes* complex (Kimani-Njogu and Overholt 1997).

The larger size of *C. typhae* relatively to the other species of the *flavipes* complex could result from an adaptation to host size, *S. nonagrioides* being a rather large noctuid relative to other Poales stem borer hosts for the *flavipes* complex. The size of a solitary parasitoid has been often reported as a plastic trait varying with host size; in gregarious parasitoids, the clutch size can be plastic and varies with host size (Godfray 1994). An evolutionary relationship between the size of gregarious parasitoids and the host size can exist if there is a genetic constraint on the clutch size, which is very likely, at least due to limits in the number of mature oocytes. The differentiation of other morphological traits may result from selective sweep or genetic correlation with other adaptive traits exposed to differential selection.

The morphological identification of species of the *flavipes* complex relies on a combination of slight differences, and their observation requires specific expertise, so a molecular diagnoses using CO1 or the virulence gene CrV1 (Dupas et al. 2006; Branca et al. 2011) remains the easiest identification method.

The geographic distribution and ecology of *C. typhae* have been reported by Kaiser et al. (2015). Here we provide evidence that the probability of collecting *S. nonagrioides* parasitized by *C. typhae* depended on the number of collected larvae. In several visited countries, this number was not sufficient to assess the presence of the wasp, so it may well be present over the sub-Saharan distribution of its noctuid host. The percentage of parasitized larvae was highly variable between localities, and even between periods of sampling in the same locality. Abundance of *C. typhae* within a locality may vary depending on the rainy season. Indeed Mailafiya et al. (2010) found that *C. sesamiae* was more abundant during the rainy season, and here (Table 6), the highest values of *C. typhae* abundance were observed in the middle of the rainy season (December), whereas lower values corresponded to the beginning of the season (Makindu-Masimba area, rainy season from November to January, Mailafiya et al. 2010). Other localities in western Kenya with rainy seasons from March to August and October to December had the highest parasitism observed in June. Regarding mean parasitism rates, about 20% of *S. nonagrioides* larvae were successfully parasitized by *C. typhae*. The same mean value or range of parasitism rates were observed in stem borers parasitized by *C. sesamiae*, and by *C. flavipes*, in maize and sorghum in Kenya (Mailafiya et al. 2010), and by *C. chilonis* in rice in China (Lou et al. 2014), but lower values were also observed (Jiang et al. 2006). This mean value is much lower than that observed in laboratory conditions. One limiting factor of parasitism success in natural conditions may be the behavior of host larvae, which hide inside the stem galleries with entrances that are naturally plugged with residues from boring, and move about mostly during the night, whereas the wasps are diurnal. Larvae also defend themselves by biting to death the wasps attempting to oviposit, killing 30-40% of them (Potting et al. 1997). Dispersion of these small parasitoids may also limit their efficiency, and would explain why mass releases of *C. flavipes* performed in sugarcane fields in Brazil successfully raised the parasitism rate to a range of 40-60% (Botelho and Macedo 2002; Dinardo-Miranda et al. 2014). Our data on *C. typhae* also show that parasitism rates as high as 70% can occur, although this rates was observed only once, and the next closest value was half lower. Even higher seasonal peaks were also reported in the case of the noctuid stem borer *Busseola fusca* parasitized by *C. sesamiae* on sorghum in South Africa (Kfir and Bell 1993), and of the crambid rice stem borer *C. suppressalis* in China (Lou et al. 2014).

Presence of *C. typhae* in different years in the same place showed that locality and plant-host combination was a good criterion for finding this new species. Very rare occurrence of parasitism of *S. nonagrioides* by *C. sesamiae and C. flavipes*, observed in less than 1% of the larvae, means that species identity has to be checked systematically.

The longevity of *C. typhae* and reproduction dynamics resemble those observed for the other species of the *flavipes* complex, which are typical short lived pro-ovogenic parasitoid wasps (Quicke 1997), i.e. females emerge with mostly mature oocytes and oviposit shortly after being mated, until egg-depletion. A literature review allows comparisons with three other species of the complex. *C. typhae* adult longevity was close to that of *C. sesamiae*, i.e. a mean longevity of about three days when fed honey at 25° and 60% RH (Sallam et al. 2002), but shorter than the longevity recorded in similar conditions for *C. flavipes* (about 5-6 days, Potting et al. 1997) and for *C. nonagriae* (about 12 days, Muirhead et al. 2008). Longevity in outdoor conditions may be longer due to cooler temperatures at night and the opportunity to rest in favorable micro-niches provided by plants. In the *flavipes* complex the dynamic of female reproduction follows their longevity, since most offspring are produced from the first two ovipositions in *C. typhae*, as in *C. sesamiae* (Sallam et al. 2002), and along 4-5 ovipositions in *C. flavipes* (Potting et al. 2007), with the exception of *C. nonagriae* which produces most offspring during the first two ovipositions, although it can live for several days. This behavior may have been selected in response to the defense behavior of stem borer noctuid larvae, which threatens female survival at each oviposition. With regard to realized fecundity, data available for the species complex were mostly the number of offspring produced from the first oviposition, which can be estimated at 60 offspring in *C. typhae*. This value is intermediate between higher value observed for *C. nonagriae* (about 90 offspring from one oviposition, Muirhead et al. 2008), and lower value observed for *C. sesamiae* and *C. flavipes* (from 25 to 45, depending on both parasitoid strain and host species, Jiang et al. 2004, Mochiah et al. 2001, NgiSong et al. 1998, Sallam et al. 2002). Altogether, these data indicate an evolution of the reproduction strategy within the *flavipes* complex. Considering longevity and oviposition dynamic, *C. typhae* appeared to be closer to *C. sesamiae* than to *C. flavipes* and *C. nonagriae*, which is in accordance with the estimated phylogenetic proximity (Muirhead et al. 2012; Kaiser et al. 2015).

The parasitism success of *C. typhae* in European host populations, assessed in the present work, was initially questioned because European *S. nonagrioides* are genetically well differentiated from African populations (Moyal et al. 2011), and they may have evolved immune responses adapted to European parasitoids and pathogens. However, the variation of reproductive success of *C. typhae* in the different host populations did not depend on the continental origin of the host, because *C. typhae* performed globally better in the Italian population than in the French and the Kenyan ones. A genetic differentiation of the Italian host population is unlikely because a recent study based on the analysis of micro-satellite markers showed an absence of genetic structure of *S. nonagrioides* collected in Europe, and in the Near and Middle East (Kader et al., unpublished data). We are more inclined to suspect that the laboratory rearing conditions of the noctuids had an effect on *C. typhae* parasitism success. Indeed, Italian larvae tested in the present work had been reared in a different laboratory than larvae from the French and the Kenyan populations. The Italian laboratory uses a different diet (Giacometti 1995), and larval food is known to influence immune response of Lepidoptera larvae (Smilanich et al. 2009; Vogelweith et al. 2015). Comparison of diets on susceptibility of the three host populations to *C. typhae* will allow this hypothesis to be tested.

In the areas where *C. typhae* have been found, in eastern sub-Saharan Africa, *S. nonagrioides* is rarely seen on maize, sorghum or sugarcane, whereas this is the case in more western parts of Africa and in Europe and the Near and Middle East. However *C. typhae* would probably parasitize *S. nonagrioides* at least on maize, if introduced for biological control, because in laboratory conditions host larvae are readily accepted when fed on maize stem and fecal pellets and eaten stem tissues are highly attractive, triggering intense behavioral examination of the host with antennal tapping.

In conclusion, this study adds a fifth species to the *Cotesia flavipes* complex. Despite the number of individual studies that illustrate the diversity of ecological adaptations in this complex, a comprehensive analysis of the *flavipes* species group is still needed. It will require the joint study of all populations across the geographical and ecological range of the *Cotesia flavipes* complex. The use of an integrative taxonomic approach (combining morphological, molecular, biological and geographical data) will be of paramount importance in recognizing and characterizing this economically important complex of parasitoid wasps. The new *C. typhae* species is an interesting potential biological control agent of the Mediterranean corn borer *S. nonagrioides*, because of its strict host-specificity to that species, at least in its native area, precluding potential negative impact on non-target host species populations.

## Supplementary Material

XML Treatment for Cotesia typhae

## Appendix

**Table:** Genebank accession numbers of CO1 sequences

Species	Genbank accession nbr	Sample name
*Cotesia chilonis*	KJ882549	P6679
	KJ882550	P6680
	KJ882551	P6681
*Cotesia flavipes*	KJ882544	P0433
	KJ882545	P0434
	KJ882546	P0435
	KJ882547	P2541
	KJ882548	P4706
*Cotesia sesamiae* Kitale	KJ882497	G4540
	KJ882501	G4594
	KJ882512	G4636
	KJ882527	G4701
	KJ882528	G4703
	KJ882529	G4708
	KJ882530	G4907
	KJ882532	G4915
	KJ882537	G5778
	KJ882543	CsK
*Cotesia sesamiae* Mombasa	KJ882495	G4511
	KJ882496	G4512
	KJ882500	G4572
	KJ882513	G4652
	KJ882533	G5699
	KJ882538	G7338
	KJ882541	Mhk
*Cotesia typhae*	KJ882502	G4608
	KJ882503	G4609
	KJ882507	G4614
	KJ882508	G4615
	KJ882510	G4618
	KJ882511	G4619
	KJ882514	G4655
	KJ882515	G4656
*Cotesia typhae*	KJ882516	G4664
	KJ882518	G4666
	KJ882519	G4667
	KJ882521	G4675
	KJ882522	G4676
	KJ882523	G4677
	KJ882531	G4909
	KJ882534	G5726
	KJ882535	G5773
	KJ882539	Mbita
	KJ882540	MbL
	KJ882542	Mkd
